# Little Homes for Ailing Children

**Published:** 1912-02-03

**Authors:** M. Stephany

**Affiliations:** Secretary Board of Guardians for Relief of Jewish Poor. 127 Middlesex Street, Bishopsgate, London, E.


*To the Editor of* The Hospital.

### LITTLE HOMES FOR AILING CHILDREN.

*To the Editor of* The Hospital.

Sir,—My attention has been drawn to an article appearing in your issue of January 6, 1912, on page 366, under the heading Byways of Medical Relief, "Little Homes for Ailing Children." I am desired to ask if you will be good enough to let me know the source whence you derived the information contained in the second paragraph of that article, as the statement is not quite in accordance with the facts. Thanking you in advance for a reply.—Yours faithfully,

M. Stephany,
Secretary Board of Guardians for Relief of Jewish Poor.

127 Middlesex Street,

Bishopsgate, London, E.,

January 15.

[We are informed that partly owing to the difficulties which present themselves in respect to food for Jewish children the home in question now admits children irrespective of creed, chiefly from the Southwark district, instead of confining its benefits, as was formerly the case, to children of Jewish parentage.—Ed., The Hospital.]

